# The PREPARE Study: Acceptability and Feasibility of a Telehealth Trimodal Prehabilitation Program for Women with Endometrial Neoplasia

**DOI:** 10.3390/curroncol32010055

**Published:** 2025-01-20

**Authors:** Elise P. Legault, Paula A. B. Ribeiro, Danielle Moreau-Amaru, Emmanuelle Robert, Sara Forte, Alain S. Comtois, Vanessa Samouëlian, François Tournoux

**Affiliations:** 1 Coeurlab Research Unit, Centre de Recherche du Centre Hospitalier de l’Université de Montréal, Montréal, QC H2X 0A9, Canada; paula.ribeiro.chum@ssss.gouv.qc.ca (P.A.B.R.); francois.tournoux.med@ssss.gouv.qc.ca (F.T.); 2 Département des Sciences de l’Activité Physique, Université du Québec à Montréal, Montréal, QC H2L 1Y4, Canada; comtois.alain-steve@uqam.ca; 3 Service de Gynécologie Oncologique du Centre Hospitalier de l’Université de Montréal, Montréal, QC H2X 0C1, Canada; danielle.moreau.ccomtl@ssss.gouv.qc.ca (D.M.-A.); emmanuelle.robert.chum@ssss.gouv.qc.ca (E.R.); vanessa.samouelian.med@ssss.gouv.qc.ca (V.S.); 4 Centre de Recherche du Centre Hospitalier de l’Université de Montréal (CRCHUM), Montréal, QC H2X 0A9, Canada; 5 Département d’Obstétrique-Gynécologie, Université de Montréal, Montréal, QC H3C 3J7, Canada; 6 Service de Cardiologie du Centre Hospitalier de l’Université de Montréal, Montréal, QC H2X 0C1, Canada

**Keywords:** cancer, exercise, preoperative exercise, diet therapy, psychosocial intervention, quality of life, anxiety, depression, hospitalization, postoperative complications

## Abstract

Patients with endometrial neoplasia (EN) often have multiple comorbidities and a higher surgical risk. Prehabilitation programs (PPs) combine various interventions to improve preoperative conditions and reduce impairment due to surgical stress. We conducted a pragmatic pilot study to evaluate the acceptability and feasibility of a trimodal telehealth PP (exercise, nutrition, and psychological support) for EN patients. The participants could select their exercise group: (1) a supervised PP (SPP), group sessions 3×/week; (2) a semi-supervised PP (SSPP), group session 1×/week, training alone 2×/week; or (3) a physical activity counseling session (PACS). Out of the 150 EN patients awaiting surgery screened during the 18 months of the study recruitment, 66% (99/150) were eligible, and 40% consented to participate (SPP, n = 13; SSPP, n = 17; PACS, n = 9). The overall dropout was low (13%; 5/39), with no significant differences across groups. No serious adverse events occurred. We observed a positive impact on different outcomes across the different groups, such as in the Functional Assessment of Cancer Therapy quality of life score (SPP; delta = 6.1 [CI: 0.9; 12.6]) and functional capacity measured using the 30″ sit-to-stand test (PACS delta = 2.4 [CI: 1.2; 3.6]). The same-day hospital leave was high in the SSPP group (54.5%). Our pilot telehealth PP seems to be safe, feasible, and well accepted and may procure clinical and patient-centered gains that need to be confirmed in a larger trial.

## 1. Introduction

Endometrial cancer is on the rise in North America, ranking among the most common cancers in women and accounting for 7.4% of new cancer cases diagnosed in 2023 [1]. Surgery remains the primary treatment for endometrial cancer [2], and postoperative morbidity is common in this population [3,4]. Operative risk and recovery are influenced not only by risk factors for endometrial cancer, such as obesity [5,6,7], diabetes [4,5], and metabolic syndrome [4,7], but also by poor physical function, low aerobic capacity, poor nutritional status, low quality of life, and depression [8,9,10,11,12]. These factors are commonly seen in women candidates for surgery for endometrial cancer [3,8,9,13].

The period between cancer diagnosis and the initiation of treatment is often stressful [14,15], and limited or no psychological support and exercise is typically offered. Interventions such as prehabilitation could be conducted during that time to reduce the impact of these risk factors and ultimately improve recovery. Prehabilitation programs combine interventions to enhance physical, metabolic, and psychosocial reserves during the preoperative period to minimize the incidence and severity of future impairment following treatments like surgery [16]. Multimodal prehabilitation programs, which primarily include exercise, have shown positive effects on surgical outcomes in individuals living with cancer undergoing abdominal surgery [17,18,19], but evidence remains limited in women with gynecological cancers [20]. Further, the effect of prehabilitation on quality of life and mental health remains unclear [18,19,20].

Previous studies typically proposed either highly supervised in-hospital interventions, home-based interventions with little or no supervision, or a hybrid of both [18,19]. However, all these interventions faced challenges, such as travel and distance barriers for in-hospital programs or lack of motivation for individuals training alone at home [21,22]. A telehealth prehabilitation program could be a third option for those unable to adhere to the current care offer, with the hope of improving program uptake and compliance. However, there may be other patient- and implementation-related challenges, such as technology access issues, lack of familiarity with this kind of platform for both participants and healthcare teams [23,24], or just the complexity of coordinating multimodal interventions among different healthcare professionals in a limited preoperative window of time [25,26].

To understand and describe challenges that may hinder future implementation of telehealth prehabilitation programs in this population, we conducted a pragmatic pilot study. The primary objective was to evaluate the acceptability and feasibility of different levels of exercise supervision in the context of telehealth trimodal prehabilitation programs in women with endometrial neoplasia awaiting surgery. The secondary objectives were (1) to describe the characteristics of participants according to their choice of group and (2) to describe potential clinical, economic, and patient-centered gains related to the intervention.

## 2. Materials and Methods

### 2.1. Study Design and Setting

**Scheme 1:**
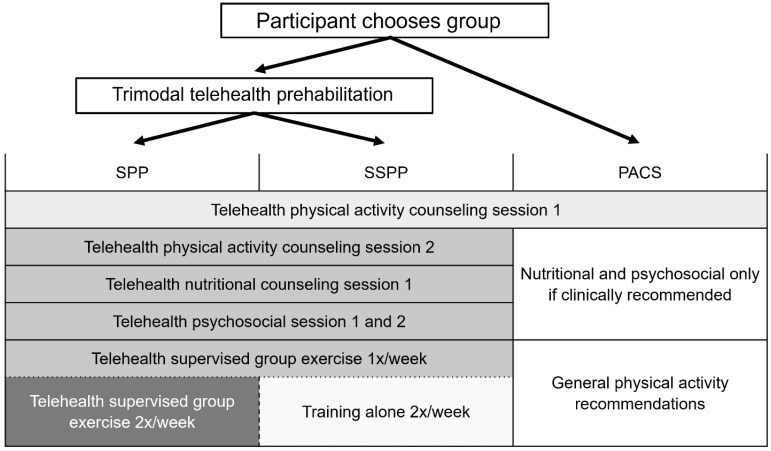
Study design and interventions. PACS, physical activity counseling session; SPP, supervised prehabilitation program; SSPP, semi-supervised prehabilitation program.

This was a pre- and post-intervention feasibility study of complex telehealth trimodal prehabilitation programs in women with endometrial neoplasia. The design of the study was pragmatic as each participant was offered a choice between three intervention groups: the supervised prehabilitation program (SPP), the semi-supervised prehabilitation program (SSPP), or the physical activity counseling session (PACS). Detailed explanations regarding each intervention were provided by phone to all eligible women, and in the written informed consent form which was sent by email. Women were then invited to select the intervention they thought was the most adapted to their needs and daily personal constraints. All assessments and interventions were delivered remotely by videoconference either using Microsoft Teams or Zoom (Zoom Video Communications, Inc., San Jose, CA, USA, 2020). Scheme 1 describes the study design and interventions.

The study was carried out at the Department of Gyneco-Oncology at the University of Montreal Hospital (Centre Hospitalier de l’Université de Montréal, CHUM), in which the standard of care is based on an Enhanced Recovery After Surgery (ERAS^®^) protocol implemented according to the international guidelines for gynecological oncology of the ERAS^®^ Society [27,28]. The ERAS^®^ protocol consists of a multimodal approach to improve the functional rehabilitation of patients after surgery and includes several items such as preoperative counseling; a standardized approach in anesthetic management and postoperative strategies to prevent nausea, vomiting, and pain; a restriction of tubes and catheters; early mobilization; and oral feeding [29]. However, the current guidelines of the ERAS^®^ Society for perioperative care in gynecologic/oncology state that although extrapolated work in colorectal surgery shows that certain patients benefit clinically from prehabilitation, further work in gynecologic oncology is needed [30].

### 2.2. Participants and Recruitment

Women diagnosed with grade 1 or 2 endometrial intraepithelial neoplasia or endometrioid adenocarcinoma awaiting surgery at the CHUM were invited to participate in the study. Exclusion criteria were as follows: (i) cardiac conditions contraindicating vigorous-intensity exercise or other medical conditions contraindicating exercise; (ii) history of myocardial infarction and/or revascularization and/or stroke without prior medical approval; (iii) severe uncontrolled anemia or hemoglobin < 90 g/L; (iv) insulin-dependent diabetes; and (v) no personal access to the internet or to a technological device with a camera (computer, smartphone, tablet). See Table S1: Inclusion and exclusion criteria in Supplementary File S1 for more details. Those who were eligible and agreed to participate received the consent form by email, and their informed consent was obtained by videoconference at the start of the baseline assessment meeting. All consent forms were collected and stored at the institution’s REDCap, a secure, web-based software platform designed to support data capture for research studies [31,32]. This study was approved by the Human Research Ethics Committee of the CHUM (Protocol #19.204).

### 2.3. Interventions

The prehabilitation programs lasted two to eight weeks depending on the time available between enrollment and surgery. The three groups differed in the level of intervention received (Scheme 1), ranging from highly supervised to moderately supervised trimodal interventions (SPP and SSPP groups), or only one physical activity counseling session (PACS group). The PACS group did not receive any nutrition or psychosocial interventions, except in cases at risk for malnutrition and/or cases with diagnosed clinical depression. The nutritional and psychological interventions were delivered by a registered dietitian and an onco-psychologist from the gyneco-oncology service. The exercise intervention was planned and delivered by the research team and kinesiologists from the Virage Foundation, a local non-profit organization affiliated with the study’s institution.

#### 2.3.1. Physical Activity Counseling Sessions

All participants received the baseline physical activity counseling session that included the following behavior change techniques [33]: education, goal setting, action planning, and self-monitoring of exercise behavior. Education included providing verbal and written information about the positive health effects of physical activity and healthy lifestyle recommendations during the cancer care continuum (prior, during, and after treatments). The information was based on the national recommendations of the American Cancer Society and the American College of Sports Medicine [34]. The participants were asked to set an exercise goal; they were supported in planning actions to reach their goal and asked to self-monitor their exercises using a journal that was provided by the research team. Participants who chose either the SPP or SSPP program had a second counseling session that included a demonstration and instructions on how to perform the exercises [33] and on how to assess their perceived level of exertion using the 10-point Borg rating of perceived exertion (RPE) scale [35]. During this counseling session, the participants were asked to assess their RPE after each exercise, and adjustments were made when the target intensity was not reached.

#### 2.3.2. Exercise Intervention

The SPP and SSPP groups differed only by the level of supervision, not the exercise prescription: the SPP group had supervised training three times per week, and the SSPP group had supervised training once per week and trained alone for the two other training sessions (Scheme 1). The intervention was home-based and virtually supervised by kinesiologists from the Virage Foundation. Exercise sessions consisted of a 10 min warm-up, 35 min of combined moderate-to-vigorous-intensity resistance and aerobic exercises, and a 10 min cool-down including stretching exercises. Exercise prescription was adapted to the participant’s baseline physical capacity, and there were up to four levels of progression that changed every two weeks. The kinesiologists regularly asked the participants to assess their RPE on the Borg scale (0–10) during and following the exercise session to ensure that they were exercising at the prescribed moderate intensity (RPE 3–4 for entry level 1) and vigorous intensity (RPE 5–6 starting at level 2). Also, the participants were asked to describe the overall RPE for each exercise session in their journal. Resistance exercises included seven functional movements (i.e., squats), each lasting 30–60 s of effort with 30–60 s of rest, that targeted the whole body in a circuit training format. The participants used their body weight, or an elastic band provided by the research team as resistance. Aerobic exercises were integrated into the resistance circuit training and consisted of three short bouts of vigorous-intensity movements using the participants’ bodyweight (e.g., jogging in place) or available equipment (stairs, stationary bike) lasting 30–60 s. The aerobic and resistance exercise circuit was performed twice. Women were also encouraged to perform an additional optional progressive training session of continuous moderate-intensity (RPE 3–4/10) aerobic exercise (walking, running, cycling, etc.). The duration of this optional training session was at least 10 min depending on the participant’s baseline physical activity level and increased up to 40 min.

#### 2.3.3. Nutritional Intervention

A 60 min counseling session was led by a specialized registered dietitian. The session aimed to provide the SPP and SSPP groups with information on the following: (1) food sources and adequate protein intake to promote a preoperative anabolic state; (2) adequate nutrition in the immediate postoperative period; and (3) healthy eating and control of the metabolic profile. The PACS group received the same type of nutritional counseling only if they were at risk of malnutrition, which was screened at baseline using the Canadian Nutrition Screening tool, comprising (1) a nonintentional loss of body mass of ≥10% in the previous six months and (2) a reduced food intake of ≥50% for more than a week [36].

#### 2.3.4. Psychosocial Intervention

The participants in the SPP and SSPP groups received two group support sessions lasting 45–60 min led by a specialized onco-psychologist. The first meeting aimed to introduce the benefits of empowerment and help women gain control by encouraging behavioral activation, identifying obstacles to behavioral activation, and developing motivation. The participants were also given strategies to identify their needs and to communicate them effectively to their loved ones through examples of what to say and how to delegate certain tasks and express their emotions related to their situation. The second meeting focused on normalizing anxiety. The participants were introduced to relaxation techniques and tools for managing stress and anxiety. The PACS group only received this psychosocial intervention if they had been diagnosed with clinical depression. This information was obtained from the participants’ electronic medical records during the baseline assessment.

### 2.4. Outcomes

The participants were assessed at baseline and following the study’s interventions within the week before surgery. Detailed descriptions of the outcomes and assessment time points are presented in Table S2 Supplementary File S1. Clinical data including comorbidities, preoperative medical assessment, and perioperative outcomes were obtained retrospectively from the participants’ electronic medical records (EMRs).

The primary outcomes of feasibility and acceptability included the following:

1. Study enrollment and dropout rates.
2. Compliance with the SPP and SSPP interventions, based on the attendance rates for the exercise sessions and the nutritional and psychosocial meetings. Compliance information was obtained from the exercise journal provided by the research team and participants’ presence sheets from the kinesiologist, the dietitian, and the psychologist. The participants were asked to record in their exercise journal their weekly participation in the prescribed exercise sessions and the exercise sessions’ overall intensity (RPE 0–10) [35].

The secondary outcomes included the following:

1. Additional feasibility and acceptability assessments were obtained from the exercise journal and included participants’ perspectives of the program and adverse events. The participants’ perspectives of the program were assessed by asking the participants to rate the exercise session enjoyment on a 10-point scale, where 0 is no enjoyment and 10 is maximum enjoyment. Adverse events (AE) were self-reported weekly by the participants in their exercise journals and characterized as “any symptoms, pain or injury related to exercise”. A trained medical practitioner analyzed each AE and categorized them according to their seriousness, relatedness (relation to the exercise intervention), and expectedness in accordance with AE reporting guidelines [37]. Seriousness was graded using Common Terminology Criteria for Adverse Events (CTCAE), version 5.0: grade, 1, mild; 2, moderate; 3, severe or medically significant; 4, life-threatening consequences; 5, death related to AE [38].
2. The participants’ clinical characteristics were obtained either by interview, validated questionnaires, or from the EMRs and included clinical, demographic, and socioeconomic data: age, smoking status, body mass index (BMI), comorbidities, transtheoretical model Stages of Change [39], self-efficacy for exercise using the French-language version of the Exercise Confidence Survey [40], and physical activity level, which was assessed by interview.
3. Clinical, economic, and patient-centered gains (Table S2 Supplementary File S1 for assessment time points):
  - Functional capacity was virtually assessed using the 30″ sit-to-stand test [41]. Improvements in functional capacity were considered clinically significant if the difference compared to baseline was equal to or greater than 2 repetitions [42].
  - The Patient-Reported Outcome Measures (PROMS) assessments included psychology and health-related quality of life assessed using two validated questionnaires: the Functional Assessment of Cancer Therapy (FACT-G, general; FACT-En, endometrial), a quality of life measure developed specifically for people with cancer [43], and the French-Canadian version of the Hospital Anxiety and Depression Scale (HADS) [44]. The participants also answered nutritional questions before and after the intervention to assess their knowledge of protein foods and their intention to change their nutritional habits. These questionnaires were completed individually (not interviewed) using an online electronic data capture tool (REDCap) [32].
  - Perioperative outcomes included the following: surgical factors (American Society of Anesthesiologists (ASA) grade (1–4), type of surgery, surgical method and duration), hospital LOS, postoperative pain perception on a visual analog scale (0–10), 30-day intensive care admission, 30-day emergency room (ER) visit, and surgical complications graded using the Clavien–Dindo classification [45].

### 2.5. Data Processing and Statistical Analysis

The SPP and SSPP trimodal prehabilitation program success was determined by satisfying the two following criteria: (1) a dropout rate of less than 20%, and (2) 67% or more of participants that successfully completed the interventions, i.e., received the nutritional counseling intervention and at least 1 psychosocial counseling intervention and completed 67% or more of the exercise training sessions (a minimum of 4–16 exercise sessions out of 6–24 depending on the preoperative period duration). Details on the exercise compliance data processing according to groups are described in Appendix A.

This study’s data was collected and managed using REDCap electronic data capture tools hosted at CHUM [31,32]. Data descriptive analysis: categorical items were reported as frequencies (n) and proportions (%), and continuous variables as mean and standard deviation (SD). Considering the small sample size, the median and interquartile range (IQR) were also reported. Individual patient data, pre–post-intervention deltas, and bias-corrected accelerated bootstrap 95% confidence intervals (BCa 95% CIs) were calculated for functional capacity, FACT-En, FACT-General (FACT-G) subscores, and HADS scores. Changes in outcomes reaching the minimum clinically important difference were described for the 30″ sit-to-stand test [42] and the FACT-G [3]. The dataset was analyzed using IBM’s SPSS Statistics^®^ (version 26.0; SPSS Inc., Chicago, IL, USA).

## 3. Results

### 3.1. Feasibility and Acceptability: Study Enrollment and Participation

**Figure 1:**
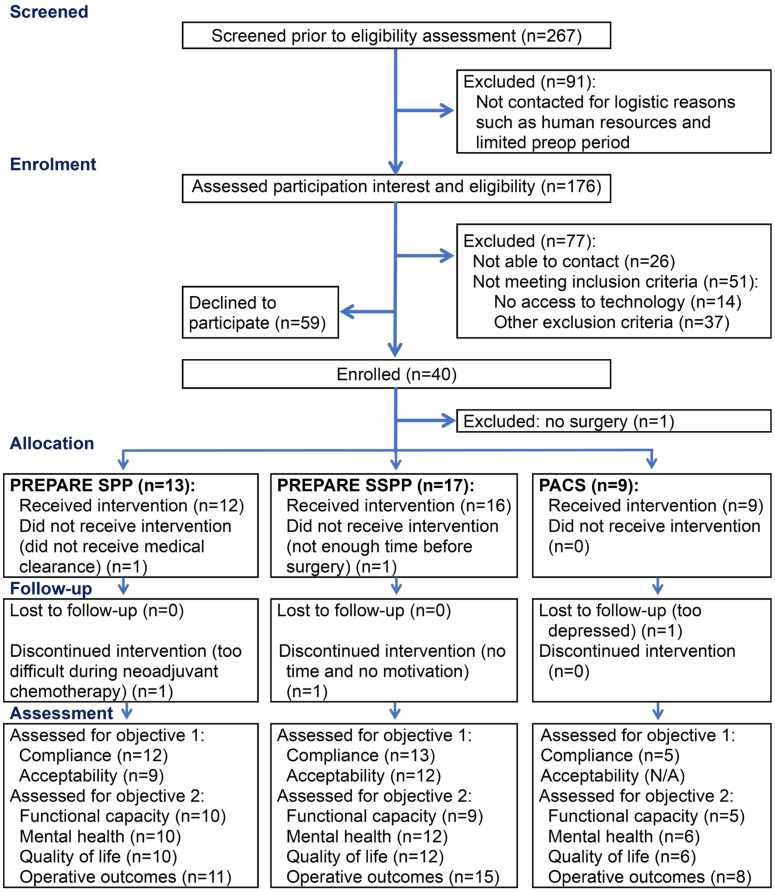
Study flow chart. N/A, not applicable; PACS, physical activity counseling session; SSPP, semi-supervised prehabilitation program; SPP, supervised prehabilitation program.

From June 2021 to February 2023, 267 women were identified by the CHUM’s gyneco-oncologists as potentially eligible for the PREPARE study. An important proportion of women (91/267, 34%) were not contacted either because of an inability to assess eligibility in the limited preop time frame, or because of a time period prior to surgery of less than two weeks. Figure 1 presents the study flow chart.

**Figure 2:**
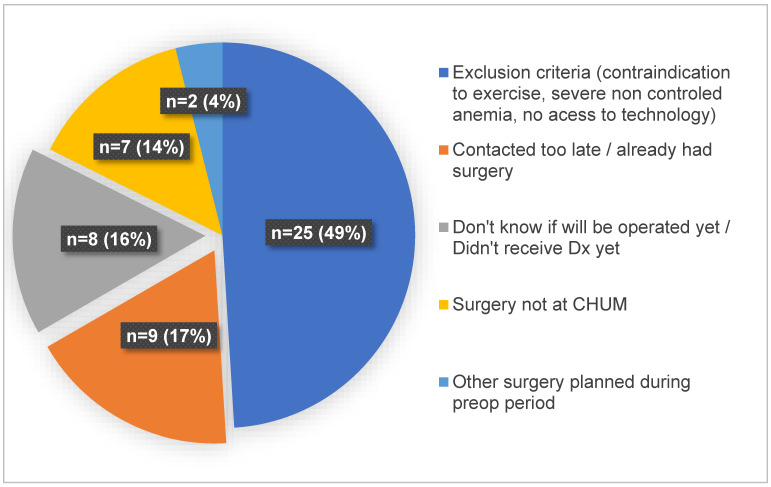
Reasons for excluding women from the study at enrollment.

**Figure 3:**
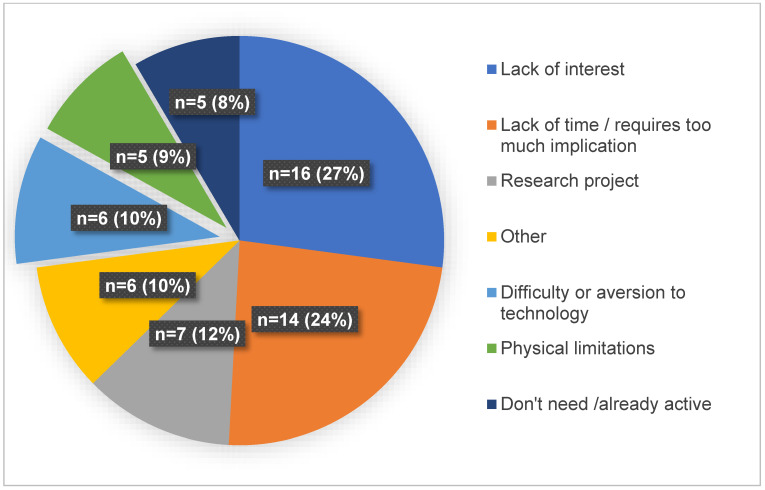
Motives for refusal to participate reported by eligible women.

Among those contacted, 66% (99/150) of the women were found to be eligible, and 40/99 consented to participate. Among the non-eligible women, 27% (14/51) were excluded from the study because they did not have access to the necessary technology required for telehealth interventions, such as an Internet connection or a computer with a camera, a tablet, or a smartphone. The reasons for excluding women from the study at enrollment are detailed in Figure 2. The motives for refusal to participate reported by eligible women are detailed in Figure 3.

The mean preoperative phase duration was 58 ± 49 days, from the baseline assessment to the day of the surgery. One participant in the PACS group received the nutritional counseling session since she was diagnosed with malnutrition, and one participant with depression was offered the psychosocial sessions but refused considering she already had regular follow-ups with her own psychologist. The mean group exercise session enjoyment score was 7.3 ± 1.8 (SPP, 8.0 ± 1.5; SSPP, 6.9 ± 1.9).

### 3.2. Participants’ Characteristics

Table 1 describes the participants’ characteristics according to group choice. The participants’ mean age was 63 ± 9 years, and they were mostly diagnosed with endometrioid adenocarcinoma (77%). Most participants (35/39, 90%) had at least one comorbidity, such as obesity (30/39, 77%), hypertension (23/39, 59%), dyslipidemia (12/39, 31%), diabetes (9/39, 23%), lung disease (10/39, 26%), cardiac/cardiovascular disease (8/39, 20%), and mental health conditions (4/39, 10%). More than a third (14/39, 36%) had musculoskeletal conditions, and only a few patients were active smokers (6/39, 15%). Women who were not reaching the physical activity recommendations tended to choose either the trimodal program that offered more supervision (SPP group) or the PACS group. More than half (7/13, 54%) of the women in the SPP group were still working full time and organized their schedule to train with the virtual exercise group three times per week in the mid-afternoon.

### 3.3. Feasibility and Acceptability

The SSPP group was chosen by 44% of the participants and was the most popular choice, as shown in the study flow chart. The total sample dropout rate was 13% (5/39), with similar dropout rates in each group (SPP, 2/13, 15%; SSPP, 2/17, 12%; PACS, 1/9, 11%), meeting our first feasibility criterion (dropout rate < 20%).

Only the SPP group reached the compliance success criterion, with 9/11 (82%) women who participated in at least two-thirds or more of the exercise training sessions and at least one of the two psychosocial interventions and attended the nutritional counseling session versus 6/13 patients (46%) in the SSPP group (see Table 2). All (8/8) of the SPP participants and 87% (7/8) of the SSPP participants complied with the exercise intervention’s prescribed intensity. For the PACS group, compliance with two-thirds or more of the weekly 180 min of leisure time physical activity was 60% (3/5).

Table S3 in Supplementary File S1 describes the characteristics of the SPP and SSPP participants according to the exercise program compliance (high vs. low). The two participants in the study that received neoadjuvant chemotherapy were unable to participate in the exercise program during their treatments. One participant reported that she no longer had the physical capacity to participate shortly after starting chemotherapy, and the other had multiple medical complications that occurred during chemotherapy and never started the exercise program.

High compliance rates were reached for nutritional counseling (overall, 23/26, 88%; SPP, 11/11, 100%; SSPP, 12/15 80%). The psychosocial intervention’s compliance rate was 60% (15/25) for attendance to the two meetings (SPP, 8/11, 73%; SSPP, 7/14 50%) and 84% (21/25) for attendance to one meeting (SPP,11/11, 100%; SSPP, 10/14 71%). Reasons for noncompliance included logistics related to the limited time prior to surgery and the common availability of the professionals and participants. One participant refused to meet with the psychologist.

### 3.4. Knowledge About Nutrition Prior to Surgery

Only 37% (11/30) of the participants (SPP 53%; SSPP 23%) completed the baseline nutritional questionnaire, and 23% (7/30) completed the post-intervention questionnaire. Therefore, only the results of the baseline assessment are reported. The mean importance attributed to nutrition before surgery on a Likert scale from 0, “not important”, to 100, “very important”, was 73 ± 17%. Seventy percent of women in the trimodal prehabilitation programs (SPP and SSPP) thought weight loss prior to surgery would be beneficial. Only 27% of the participants were able to correctly distinguish protein-rich foods from non-protein foods prior to the nutritional intervention. Most participants (82%) who answered the questionnaire intended to make changes to their eating habits, and 27% had already made some recent changes.

### 3.5. Safety of the Exercise Intervention

No serious AE related to the exercise intervention occurred during this study. Mild AE, mostly musculoskeletal pain or discomfort not limiting participation in the exercise intervention, occurred in 52% of the trimodal prehabilitation program participants, with a slightly higher proportion in the SSPP (7/12, 58%) group compared to the SPP group (4/9, 44%). One severe AE occurred in the PACS group (severe knee pain related to arthrosis). Details about AE are provided in Tables S4 and S5 in Supplementary File S1.

### 3.6. Clinical, Economical, and Patient-Centered Gains

**Figure 4:**
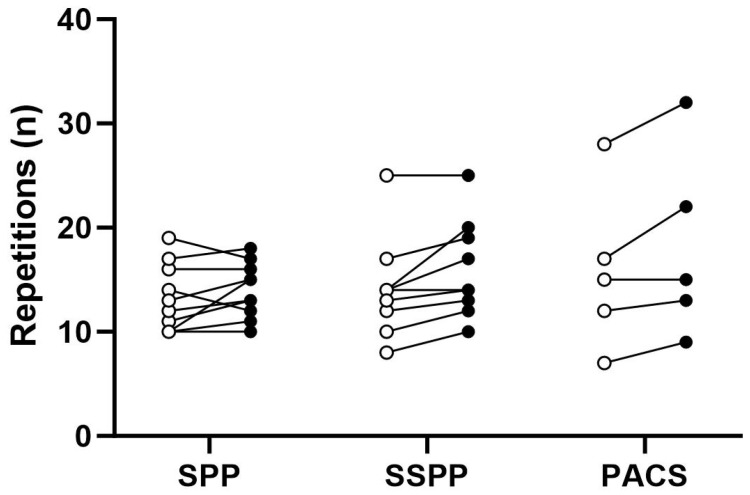
Changes in 30″ sit-to-stand test before and after the intervention according to group. Each point represents a participant, where open dots are baseline measures and filled dots are post-intervention measures. PACS, physical activity counseling session, SPP, supervised prehabilitation program; SSPP, semi-supervised prehabilitation program.

Figure 4 presents pre–post-intervention functional capacity during the preoperative period. The minimum clinically important difference in functional capacity was reached by 30% (while 20% had a clinically significant decrease) in the SPP group, 55% in the SSPP group, and 60% in the PACS group. Table S6 in Supplementary File S1 describes the mean ± SD and mean difference (95% CI) for functional capacity and PROMS.

**Figure 5:**
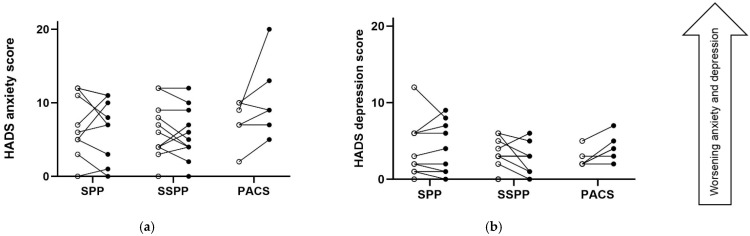
Changes in Hospital Anxiety and Depression Scale (HADS) before and after the intervention according to group; (**a**) describes results from the HADS anxiety subscale, and (**b**) describes results from the HADS depression subscale. A higher score denotes a worse condition. Each point represents a participant, where open dots are baseline measures and filled dots are post-intervention measures. PACS, physical activity counseling session; SPP, supervised prehabilitation program; SSPP, semi-supervised prehabilitation program.

**Figure 6:**
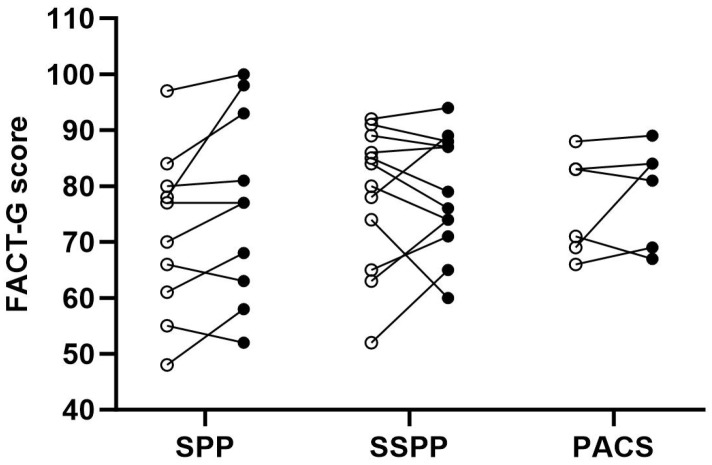
Changes in quality of life scores before and after the intervention according to group. A higher score denotes better quality of life. Each point represents a participant, where open dots are baseline measures and filled dots are post-intervention measures. FACT-G, Functional Assessment of Cancer Therapy—General; PACS, physical activity counseling session; SPP, supervised prehabilitation program; SSPP, semi-supervised prehabilitation program.

Figure 5 and Figure 6 describe the pre–post-intervention PROMS during the preoperative period. Pre–post-intervention anxiety and depressive symptoms measured with the HADS tended to increase (get worse) in the PACS group, whereas for the SPP and SSPP participants, their anxiety and depressive symptoms tended to stay the same (Figure 5 and Table S6 in Supplementary File S1).

The changes in quality of life (FACT) before and after the intervention are presented in Figure 6. The participants in the SPP group tended to have an improved quality of life following the intervention (Table S6 in Supplementary File S1). In the SSPP and PACS groups, there seemed to be no difference in pre–post-intervention general quality of life and a slight decrease in the endometrial cancer symptoms FACT subscale. Considering the functional well-being subscale (FWB) of the FACT, there was a slight increase reaching the minimum clinically important difference in the SPP group (Table S6 in Supplementary File S1).

The participants’ medical and operative characteristics are presented in Table S7 in Supplementary File S1 and do not seem to differ across groups. Table 3 describes the perioperative outcomes according to group (per protocol). Half of the women in the SPP (54%) and SSPP (47%) groups left the hospital on the day of the surgery, with only one (12%) doing so in the PACS group. Other perioperative outcomes do not seem to differ according to group choice.

## 4. Discussion

This pragmatic pre- and post-intervention pilot study showed that the PREPARE programs for women with endometrial neoplasia are safe, feasible, and well accepted. A high proportion of women with endometrial neoplasia (66%) were eligible for this type of intervention in our center. The participants had the necessary technology and were willing to use it to exercise at home under virtual supervision and to receive psychosocial and nutritional support in preparation for surgery by videoconference. Furthermore, our study showed improvements in quality of life (SPP group) and functional capacity (SSPP and PACS groups), and nearly four times more women were granted same-day postoperative hospital leave in the SPP and SSPP groups compared to the counselling-only group (PACS group). To our knowledge, this is the first study to deliver a patient-choice design, fully online, and trimodal prehabilitation for women diagnosed with endometrial neoplasia.

### 4.1. Feasibility and Acceptability

Enrollment in prehabilitation studies can be complex since participants must be contacted shortly after receiving their surgery confirmation and have limited time prior to surgery [26,46,47]. Some previous prehabilitation pilot studies have concluded that it was not feasible in certain clinical settings because of this issue [48]. In our study, an important proportion of women (91/267, 34%) were not contacted because of logistic reasons, such as the limited time frame and human resources. Nevertheless, this study was feasible considering the high proportion of women that were eligible for the program, and that 40% agreed to participate, which is comparable to enrollment rates reported in other studies [49]. The pragmatic design of the study, such as having few inclusion and exclusion criteria and tailoring parts of the exercise intervention to the participants’ preferences and needs, also using in-hospital resources to deliver the intervention, may have contributed to the feasibility success [50,51]. These design aspects also increase the programs’ potential for implementation [50,51]. Furthermore, one-third (17/51, 33%) of the non-participants could potentially be enrolled by making some adjustments, such as having referrals directly made by the clinical team and by being mindful of the timing to contact women for enrollment (i.e., optimizing internal processes by contacting patients as soon as possible after confirmation of surgery or being invited on site after meeting with the gyneco-oncologist). In our study, women were contacted by the research team, but we believe that if this program were to be recommended directly by the clinical team, retention and compliance rates could be improved [52,53].

In general, women with endometrial cancer often have multiple comorbidities, a sedentary lifestyle, and competing demands for time (i.e., work–family balance) [22,54,55]. As such, these women face greater exercise program compliance issues than the general population [56]. Despite this, the compliance and dropout rates in our study are comparable to those reported in other prehabilitation interventions for individuals living with different types of cancer, which ranged from 16 to 100% and 10 to 13%, respectively [17,18,19,49]. In fact, directly supervised hospital-based prehabilitation programs generally have higher compliance rates than home-based non-supervised programs [18,57]. However, the former are less accessible due to the distance to the hospital and related costs (i.e., parking), which are barriers to participation [22,58]. Our telehealth program has the advantages of permitting remote supervision and being accessible to women who live far from specialized hospital centers. As expected, higher compliance rates in our study were found in the group with more supervision compared to the group with lower supervision. Despite this, both groups showed improvements in clinical outcomes and PROMS, with some reaching the minimum clinically important difference.

### 4.2. Clinical, Economical, and Patient-Centered Gains

The participants in our study had similar baseline characteristics, including functional capacity and quality of life measures. The only apparent difference was the baseline moderate to vigorous weekly physical activity level, which was higher in the SSPP group. Regarding changes in functional capacity, a lower proportion of women reached the MICD in the SPP group (30%), despite their high compliance with the intervention (training sessions and intensity), compared to the SSPP group (55%). The response to exercise stimuli is multifactorial and related to training parameters (intensity, frequency, duration, and modality) and non-training parameters, such as individual characteristics (genetics, age, baseline capacity in a sedentary state, etc.), and other behaviors or environmental factors (diet, sleep, other habitual physical activities, etc.) [59,60,61,62]. Considering that women in the SPP group had lower baseline physical activity, a higher volume of exercise (i.e., more weeks of training or a higher weekly training frequency) was maybe required for some of them to reach the minimum clinically important difference. Furthermore, considering the small sample size in the PACS group and the inclusion of an outlier (a previously highly active participant), the improvements seen in this group should be interpreted with caution. In summary, the overall proportion of women in our study reaching the minimum clinically important difference in our functional capacity assessment (i.e., sit-to-stand test) (30–60%) is comparable to other cancer prehabilitation studies, with 33–84% of participants reaching the minimum clinically important difference for the 6-Minute Walk Test [18]. Furthermore, the women in our prehabilitation programs showed no declines in self-reported physical and functional well-being and even had improved general quality of life (SPP group). These results might suggest that our program was important in preventing the accelerated decline in self-reported physical function following the cancer diagnosis, as previously demonstrated [63].

Anxiety and depressive symptoms in the PACS group tended to increase, whereas there seemed to be no change (depressive symptoms) or reduction (anxiety) in the supervised and semi-supervised groups. The preoperative period is a psychologically challenging period for individuals living with cancer [64]. Women with endometrial cancer experience worse anxiety, fatigue, sleep disturbances, and pain compared to the general population during this period [15]. Psychological distress may be related to uncertainty about upcoming treatments, not knowing when they will receive their surgery, and the fear related to the cancer diagnosis [64]. Women also report a sense of loss of control [64]. Getting physically and emotionally ready to face treatment by exercising, as well as receiving professional nutritional and psychosocial counseling, may have contributed to empowering the women and thus contributed to preventing the escalation of anxiety and depressive symptoms during this period. Previous studies have found positive effects of psychological or multimodal prehabilitation on patient-reported psychological outcomes and quality of life in cancer patients [18,47,65]. However, evidence is lacking for women with gynecological cancers [20]. Larger trials would be necessary to confirm these benefits in this population.

There were no obvious discrepancies between groups regarding the ASA index (surgical risk index) or other factors that can influence surgical outcomes, such as age or surgical characteristics, including the approach and surgery duration [4,66]. Interestingly, there was a higher proportion of same-day hospital leave in the SPP and SSPP groups compared to the PACS group. We believe that preconditioning could favor early mobilization and, subsequently, early discharge from the hospital.

Our study has some limitations that need to be highlighted. First, there was a lack of a true control group. Given the well-established benefits of exercise for cancer patients and the emerging evidence supporting prehabilitation, we felt it would not be appropriate to have a group without providing at least minimal physical activity counselling. Therefore, we sought to design a study that would offer a choice among various programs, including one with just counselling, giving patients the chance to participate in designing their own intervention. Furthermore, although we conducted a more robust analysis (bootstrapped CI) that is less dependent on the sample size, our pilot results must be taken cautiously considering the small sample size, especially in the PACS group. There was also a high proportion of missing data for the functional capacity measure following the intervention in the SSPP group, which may have influenced the results in this group. However, additional analysis did not show significant differences in characteristics between the SSPP participants who completed the follow-up assessment and those who did not (such as age, baseline physical activity level, number of comorbidities, compliance with the number of exercise sessions, and compliance with the exercise intensity), as described in Supplementary File S1. Finally, considering these limitations, the changes seen in our study need to be confirmed in a larger trial.

## 5. Conclusions

Based on our feasibility findings, we suggest implementing a continuum of telehealth interventions, ranging from minimal counseling and education to more intensive, supervised multimodal approaches. This strategy could optimize accessibility, retention, and compliance rates. Telehealth prehabilitation programs have the potential to improve the care experience and quality of life of women with endometrial neoplasia awaiting surgery and shorten hospital stays. However, these findings should be confirmed in a larger study. Overall, our results provide important insights for the future implementation of preoperative telehealth programs.

## Figures and Tables

**Table 1:** Participants’ characteristics according to group choice.

	SPP (n = 13)	SSPP (n = 17)	PACS (n = 9)
**Age**, y	61 ± 7	65 ± 9	63 ± 9
	60 [56–68]	64 [59–73]	59 [54–74]
**Work status**, n (%)			
Full time	7 (54)	7 (41)	5 (56)
Part time/pre-retirement	2 (15)	2 (12)	1 (11)
Retired	4 (31)	8 (47)	3 (33)
**Smoking status**, n (%)			
Current smoker	3 (23)	1 (6)	2 (22)
Never smoked	7 (54)	13 (76)	7 (78)
Former smoker	3 (23)	3 (18)	0
**BMI**, kg/m^2^	35.5 ± 7.5	30.2 ± 5.9	33.6 ± 9.8
	37.2 [29.8–41.4]	31.1 [24.5–34.2]	36.6 [23.0–41.9]
**Comorbidities** *, n	3 ± 2	3 ± 2	3 ± 3
	3 [1.5–4]	3 [2–4]	3 [0.5–5]
**Moderate to vigorous physical activity**,	78 ± 88	246 ± 290	92 ± 143
minutes per week	40 [0–139]	176 [62–311]	80 [0–105]
≥150 min, n (%)	3 (23)	10 (62)	1 (11)
Missing, n	0	1	0
**30″ sit-to-stand test**			
Repetitions, n	12.5 ± 3.1	14.5 ± 4.9	14.2 ± 5.9
RPE, Borg 0–10	3.0 ± 0.9	2.8 ± 0.8	2.9 ± 1.5
**HADS scores**			
Anxiety, range 0–21	7.7 ± 4.2	6.7 ± 3.7	8.5 ± 5.5
	7 [5–12]	7 [4–9]	8 [4–10]
Depression, range 0–21	4.2 ± 3.4	3.6 ± 2.8	4.1 ± 3.8
	3.5 [1.2–6]	3 [1.5–5.5]	2.5 [2–4.7]
Missing, n	1	0	1
**FACT scores**			
FACT-G, range 0–108	71 ± 14	[0–13], 977 ± 12	73 ± 14
	72 [59–79]	78 [65–87]	74 [67–83]
FACT-En, range 0–172	119 ± 20	131 ± 16	122 ± 26
	123 [100–133]	133 [114–145]	129 [121–138]
Missing, n	1	0	1

Continuous variables are presented as mean ± standard deviation or median [interquartile range]. * Hypertension, dyslipidemia, diabetes, obesity, arthritis or osteoporosis, cardiovascular diseases, respiratory diseases, mental health conditions. BMI, body mass index; PACS, physical activity counseling session; RPE, rating of perceived exertion; SSPP, semi-supervised prehabilitation program; SPP, supervised prehabilitation program; y, years.

**Table 2:** Compliance with the trimodal prehabilitation program.

	SPP (n = 11)	SSPP (n = 15)	Total SPP and SSPP (n = 26)
**Compliance with exercise sessions,** n High (≥2/3) Low (<2/3) Missing, n	9 (82) 2 (18) 0	7 (54) 6 (46) 2	16 (67) 8 (33) 2
**Exercise session attendance rate,** % of total sessions	80 ± 15 83 [67–96]	73 ± 30 90 [54–98]	76 ± 24 86 [61–96]
**Mean exercise session intensity,** Borg scale 0–10 Group training Missing, n Training alone Missing, n	4.9 ± 1.1 4.6 [4.0–5.6] 3 N/A	4.6 ± 2.0 4.5 [3.0–6.0] 4 4.0 ± 1.4 4.5 [2.6–4.8] 6	4.7 ± 1.7 4.6 [3.9–6.0] 7 4.2 ± 1.4 4.5 [3.0–5.1] 6
**Compliance with nutritional and psychosocial interventions,** n Missing, n	11 (100) 0	10 (67) 0	21 (81) 0
**Compliance with trimodal intervention (trimodal PP success criterion),** n Missing, n	9 (82) 0	6 (46) 2	15 (62) 2

Continuous variables are presented as mean ± standard deviation or median [interquartile range]. N/A, not applicable; SSPP, semi-supervised prehabilitation program; SPP, supervised prehabilitation program.

**Table 3:** Perioperative outcomes according to group (per protocol).

	SPP (n = 11)	SSPP (n = 15)	PACS (n = 8)	All Participants (n = 34)
**Hospital LOS, days** Same-day leave, n (%) Next-day leave or greater, n (%)	0 [0–1] 6 (54.5) 5 (45.5)	1 [0–2] 7 (47) 8 (53)	1 [1–3] 1 (12.5) 7 (87.5)	1 [0–2] 14 (41) 20 (59)
**Postoperative pain, 0–10**	5.55 ± 2.84 5 [4–7]	4.53 ± 1.77 5 [3–6]	5.83 ± 3.66 5 [4–9]	5.13 ± 2.55 5 [4–6]
**Patients suffering from ≥1 postoperative complication, n (%)**	1 (9)	2 (13)	1 (12)	4 (12)
**Total number of complications, n (%)**	1 (9)	3 (20)	2 (25)	6 (18)
**Grade of complication (Clavien–Dindo), n** Grade 1 Grade 2	1 0	2 1	2 0	5 1
**30-day postop ER visit, n (%)**	0	2 (13)	1 (12)	3 (9)
**30-day postop hospital readmission, n (%)**	0	0	0	0

Continuous variables are presented as mean ± standard deviation or median [interquartile range]. ER, emergency room; LOS, length of stay; PACS, physical activity counseling session; SSPP, semi-supervised prehabilitation program; SPP, supervised prehabilitation program.

## Data Availability

The raw data supporting the conclusions of this article will be made available by the authors upon request.

## Supplementary Materials

The following supporting information can be downloaded at: https://www.mdpi.com/article/10.3390/curroncol32010055/s1, Table S1: Inclusion and exclusion criteria; Table S2. Description of outcome measures and assessment time points; Table S3: The characteristics of the SPP & SSPP participants according to the exercise program compliance; Table S4. Adverse events according to group; Table S5. Description of exercise related adverse events that were not expected according to group; Table S6. Change in functional capacity and patient reported outcome measures pre- and post-intervention according to group (per-protocol); Table S7. Participants’ medical and operative characteristics according to group. References [35,39,40,41,43,44,45] are cited in the supplementary materials.

## Appendix A

### Data Processing

The exercise compliance for the SPP and SSPP groups was determined by calculating the proportion of training sessions performed relative to the exercise prescription: [n of exercise sessions performed/(3 weekly sessions * n weeks prior to surgery)], where the n of weeks corresponds to the number of days between the second physical activity counseling session (exercise familiarization) and the surgery. When the preoperative period lasted longer than the 8 weeks of planned intervention, participants were permitted to continue training with the group, but their compliance was calculated based on the first 8 weeks of the intervention (to control the time effect and guarantee comparability among the participants). For the PACS group, successful compliance with the recommendations was determined by performing at least 67% of 180 min of leisure time physical activity, which would be equivalent to the success threshold for the SPP and SSPP groups (67% of the training prescription of 60 min sessions, three times per week).
