# Supplementary material for: The PREPARE Study: Acceptability and Feasibility of a Telehealth Trimodal Prehabilitation Program for Women with Endometrial Neoplasia
Source: Curr Oncol. 2025 Jan 20;32(1):55. doi: 10.3390/curroncol32010055 (PMC11763516; doi:10.3390/curroncol32010055)
Supplement: Supplementary file 1 [file curroncol-32-00055-s001.zip › curroncol-3384336-supplementary.pdf]

Supplementary File S1:

THE PREPARE STUDY: ACCEPTABILITY AND FEASIBILITY OF A TELEHEALTH MULTIMODAL PREHABILITATION PROGRAM FOR ENDOMETRIAL CANCER PATIENTS
Legault et al.

Method section supplementary information

Table S1: Inclusion and exclusion criteria

Inclusion criteria	Exclusion criteria
• Patient with grade 1 or 2 endometrial intraepithelial neoplasia or endometrioid adenocarcinoma, awaiting surgery at the University of Montreal Hospital.	• Cardiac conditions contraindicating high-intensity exercise: ischemia, unstable angina, symptomatic aortic stenosis, pacemaker, uncontrolled arrhythmia with hemodynamic symptoms, uncontrolled hypertension > 180/100mmHg, thrombophlebitis.<br>• History of infarction and/or revascularization or stroke without prior medical approval.<br>• Severe uncontrolled anemia or hemoglobin < 90g/l<br>• Insulin-dependent diabetes.<br>• Medical conditions contraindicating exercise: severe respiratory insufficiency, pulmonary embolism, severe chronic renal insufficiency, cirrhosis.<br>• No personal access to the internet or to a technological device with a camera (computer, smartphone, tablet).*

*Participants that were excluded based on this criterion were referred to the Virage Foundation to receive services (telephone based or in person).

Table S2. Description of outcome measures and assessment time points

Outcome measures and assessment method	Time points			
	Prior to enrolment	Baseline	Pre-op & post-intervention	Post-op
**Study enrolment**<br>Ratio of the n of participants that accepted / n of participants that were eligible.	x			
**Study dropout rate**<br>Ratio of the n of participants that did not receive or discontinued the intervention / n of participants enrolled.		x		

**Compliance to interventions**<br>Attendance rate to the exercise sessions, the nutritional and the psychosocial meetings (n of session completed / total n of sessions planed in the interventions).			x	
**Adverse events (AE)**<br>Symptoms, pain or injury that occurred because of the exercises. Self-reported by the participants each week in the exercise journal.			x	
**Participants characteristics**<br>By interview:<br>- smoking status, working status, physical activity level*<br><br>From EMR:<br>- Body mass index<br>- comorbidities from the preoperative medical assessment.<br><br>By validated questionnaires:<br>- transtheoretical model Stages of Change Questionnaire [1]<br>- self-efficacy assessed using the French-language version of the Exercise Confidence Survey [2]	x			
**Clinical outcomes**<br>From telehealth assessment:<br>- functional capacity assessed using the 30” sit-to-stand test [3]. Dining table chair was used, participants were instructed to use the same chair at reassessment.<br>**PROMS**<br>By validated questionnaires:<br>- quality of life using the FACT-En [4]<br>- depression and anxiety using the HADS [5]<br>By questionnaire created by research team:<br>- nutrition questionnaire (knowledge about protein foods and intention to change, etc.)		x	x	
**Perioperative outcomes**<br>From EMR:<br>- surgical factors (type of surgery surgical method and duration)<br>- hospital length of stay				X

- postoperative pain perception on a visual analog scale (0-10)<br>- 30-day intensive care admission<br>- 30-day emergency room visit surgical complication graded using the Clavien-Dindo classification [6]				

BMI, body mass index; EMR, Electronic medical records; FACT-En, Functional Assessment of Cancer Therapy – Endometrial; HADS, Hospital anxiety and depression scale; n, number; PROMS, patient-reported outcomes measures.

*Participants were asked to describe the average weekly physical activity they performed in the previous two weeks in terms of frequency, duration, and intensity using the 10-point Borg rating of perceived exertion scale (RPE) [7] and type of activity (ex. walking, gardening, sports, resistance training, etc.). Baseline physical activity level was calculated by multiplying weekly frequency and duration of activities reported by the participants that were described as moderate to vigorous intensity on Borg RPE scale.

Result section supplementary information

Table S3: The characteristics of the SPP & SSPP participants according to the exercise program compliance

	High compliance (n=16)	Low compliance (n=8)	Drop-out (n=2)
**Age,** y	60 ± 7<br>59 [55-66]	66 ± 9<br>65 [60-73]	63 ± 4<br>60 & 63
**Work status,** n (%)			
Full time	**8 (50)**	3 (37)	2 (100)
Part time/pre-retirement	3 (19)	1 (12)	0
Retired	5 (31)	**4 (50)**	0
**BMI class,** n (%)			
Normal or overweight	3 (19)	3 (37)	0
Obesity class ≥ 1	**13 (81)**	**5 (62)**	2 (100)
**Comorbidities*,** n	2 ± 1<br>2.5 [1-4]	4 ± 3<br>4 [2-6]	4 & 4
**Baseline moderate to vigorous physical activity,** minutes per week	125 ± 96<br>122 [40-195]	248 ± 384<br>110 [10-291]	0 & 157
≥150 min, n (%)	7 (44)	4 (50)	1 (50)
Missing, n	1	0	0
**Mental health conditions,** n (%)			
Yes	4 (25)	0	1 (50)
No	**12 (75)**	**8 (100)**	1 (50)

**Cancer grade (FIGO),** n (%)<br>X or 1<br>2 or 3	 **12 (75)**<br>4 (25)	 **5 (62)**<br>3 (37)	 1 (50) (grade 1)<br>1 (50) (grade 3)
**Neoadjuvant chemotherapy,** n (%)<br>Yes<br>No	 0<br>**16 (100)**	 1 (12)<br>**7 (87)**	 1 (50)<br>1 (50)
**Stage of change,** n (%)<br>Precontemplation<br>Contemplation<br>Preparation<br>Action<br>Maintenance	 0<br>2 (12)<br>4 (25)<br>2 (12)<br>**8 (50)**	 0<br>1 (12)<br>**5 (62)**<br>0<br>2 (25)	 0<br>1 (50)<br>0<br>0<br>1 (50)
**Adverse events of the intervention,** n (%)<br>Yes<br>No<br>Missing, n	 **9 (64)**<br>5 (36)<br>2	 2 (40)<br>**3 (60)**<br>3	 0<br>2 (100)
**Appreciation****, score (0-10)	7.4 ± 1.9<br>7.3 [6.2-9.4]	6.8 ± 1.5<br>6.7 [5.4-8.3]	8.4 ± 1.9<br>7.0 & 8.4

BMI, Body mass index; Y, years. Continuous variables are presented as mean ± standard deviation or median [interquartile range]. In grey, participants' characteristics that tend to differ between high and low compliance participants (no statistics were calculated considering the small sample size). Highest proportions are presented in bold characters. *Hypertension, dyslipidemia, diabetes, obesity, arthritis or osteoporosis, cardiovascular diseases, respiratory diseases, mental health conditions. **Appreciation score ranges from 0 (no enjoyment) to 10 (high enjoyment).

Among the participants with low compliance to exercise in the trimodal prehabilitation groups, half were retired, most (62%) were in the "preparation" stage of change for physical activity and 40% experienced adverse events (vs. 64% for participants with high compliance). Also, participants with low compliance had twice as many comorbidities compared to the participants with high compliance to intervention. Participant with low compliance had a baseline moderate to vigorous physical activity level that was nearly two times higher than individuals in the compliant group. Perhaps the former were less compliant to the intervention because they were already active and did not feel the need, or have the time, to do more exercises.

Table S4. Adverse events according to group

	SPP (n=9)	SSPP (n=12)	PACS (n=5)
**Total number of AE, n**	11	14	3
**Severity (grade 1 to 5)**			
Grade 1	11	14	2
Grade 2	0	0	0
Grade 3	0	0	1
**Total number of AE related to exercise intervention, n**	9	11	N/A
Not expected, n	1	2	
Participants with ≥1 AE related to exercise intervention, n (%)	4 (44)	7 (58)	

AE, adverse events; N/A, non-applicable. PACS, physical activity counseling session; SPP, supervised prehabilitation program; SSPP, semi-supervised prehabilitation program. Grade, 1, mild; 2, moderate; 3, severe or medically significant; 4, life-threatening consequences; 5, death related to AE. All events related to exercise intervention were mild (grade 1). Grade 3 event was described as knee pain following 2 hours of shopping.

Table S5. Description of exercise related adverse events that were not expected according to group

SPP	SSPP
- “Mal au talon et mollet probablement dû à l'étirement”	- “Slight pain in the knee when standing up.”<br>- “Étiré muscle de l'aine droite et mal de hanche”

SPP, supervised prehabilitation program; SSPP, semi-supervised prehabilitation program

Table S6. Change in functional capacity and patient reported outcome measures pre- and post-intervention according to group (*per-protocol*)

	SPP (n = 11)			SSPP (n = 15)			PACS (n = 8)		
	Baseline Mean ± SD	Post Mean ± SD	Mean dif. (95% CI)	Baseline Mean ± SD	Post Mean ± SD	Mean dif. (95% CI)	Baseline Mean ± SD	Post Mean ± SD	Mean dif. (95% CI)
**30” Sit-to-stand**, number of repetitions	13.2 ± 3.2	14.0 ±2.6	0.8 (-0.3, 1.9)	14.1 ± 4.8	16.0 ±4.7	**1.9 (1.0, 3.0)**	15.8 ±7.8	18.2 ± 9.0	**2.4 (1.2, 3.6)**
Missing, n	1	1	1	6	6	6	3	3	3
**FACT-En (0-172)**	120 ± 20	126 ±23	**6.1 (0.9, 12.6)**	134 ± 15	129 ±17	-4.5 (-9.6, -0.0)	131 ±10	130 ± 12	-1.1 (-6.6, 6.7)
EC subscale (0-64)	48 ± 7	49 ± 8	1.0 (-1.3, 3.8)	55 ± 4	51 ± 9	**-4.8 (-8.8, -1.5)**	54 ± 4	51 ± 4	**-3.4 (-6.2, -0.5)**
**FACT-G (0-108)**	71 ± 15	77 ± 17	**5.2 (1.6, 9.9)**	78 ± 12	78 ± 11	0.3 (-4.5, 5.6)	77 ± 9	79 ± 9	2.3 (-1.8, 8.0)
Physical (0-28)	22 ± 3	22 ± 4	-0.5 (-2.5, 1.4)	24 ± 5	24 ± 4	-0.1 (-1.8, 1.8)	22 ± 6	23 ± 3	0.8 (-2.2, 5.6)
Social/family (0-28)	18 ± 6	20 ± 7	**2.4 (0.6, 4.5)**	19 ± 3	20 ± 3	0.8 (-1.2, 2.7)	20 ± 4	23 ± 3	2.8 (-0.4, 5.7)
Emotional (0-24)	15 ± 4	18 ± 4	**2.1 (0.9, 3.3)**	17 ± 4	17 ± 3	0.0 (-1.8, 2.3)	16 ± 4	14 ± 5	-1.7 (-5.0, 1.7)
Functional (0-28)	16 ± 6	17 ± 6	1.2 (-0.6, 3.5)	18 ± 4	18 ± 4	-0.5 (-1.7, 0.7)	18 ± 3	19 ± 4	0.3 (-2.0, 4.0)
Missing, n	1	1	1	3	3	3	2	2	2
**HADS scores***									
Anxiety (0-21)	7.3 ± 4.3	6.9 ± 4.2	-0.4 (-2.4, 1.9)	6.1 ± 3.6	5.8 ± 3.4	-0.2 (-1.4, 0.9)	7.5 ±3.0	10.5 ± 5.4	**3.0 (0.2, 7.0)**
Depression (0-21)	3.9 ± 3.6	3.8 ±3.5	-0.1 (-1.1, 0.8)	2.9 ± 2.1	2.1 ± 2.1	-0.8 (-1.7, 0.0)	2.7 ±1.2	4.2 ± 1.7	**1.5 (0.7, 2.3)**
Missing, n	1	1	1	3	3	3	2	2	2

EC, endometrial cancer; FACT, Functional Assessment of Cancer Therapy (G, general; En, endometrial); HADS, Hospital anxiety and depression scale; PACS, physical activity counseling session; SSPP, semi-supervised prehabilitation program; SPP, supervised prehabilitation program. Values in bold represent trends toward significant difference. *Higher score denotes worse anxiety or depressive symptoms.

Considering the high proportion of missing data in the SSPP group with regards to functional capacity, additional analyses were performed to determine if the participants with missing data had different characteristics that could have influenced the results. No important differences were found between participants with missing data compared to participants that performed the post-intervention assessment for: age, baseline physical activity level, comorbidities, compliance to number of exercise sessions, and compliance to exercise intensity.

Table S7. Participants' medical and operative characteristics according to group

	SPP<br>(n = 13)	SSPP<br>(n = 17)	PACS<br>(n = 9)	All participants<br>(n = 39)
**Treatments, n (%)**				
Neoadjuvant	1 (8)	1 (6)	0	2 (5)
**ASA index, n (%)**				
II	**8 (61)**	**12 (71)**	**6 (67)**	**26 (67)**
III	5 (38)	5 (29)	3 (33)	13 (33)
**Cancer grade FIGO (2009), n (%)**				
x	0	4 (23)	2 (22)	6 (15)
1	**9 (69)**	**7 (41)**	**6 (67)**	**22 (56)**
2	1 (8)	2 (12)	1 (11)	4 (10)
3	3 (23)	4 (23)	0	7 (18)
**Cancer stage**				
Precancer	0	2 (14)	0	2 (6)
IA	**10 (77)**	**5 (36)**	**5 (71)**	**20 (59)**
IB	0	1 (7)	**2 (29)**	3 (9)
II	1 (8)	0	0	1 (3)
IIIA	0	1 (7)	0	1 (3)
IIIC	**2 (15)**	2 (14)	0	4 (12)
IVB	0	**3 (21)**	0	3 (9)
**Type of surgery, n (%)**				
Total hysterectomy	**13 (100)**	**16 (94)**	**9 (100)**	**38 (97)**
Bilateral or unilateral salpingo-ovariectomy	**13 (100)**	**16 (94)**	**9 (100)**	**38 (97)**
Sentinel lymph node dissection	11 (85)	13 (76)	8 (89)	32 (82)
Pelvic lymphadenectomy	1 (8)	3 (18)	0	4 (10)
Other	6 (46)	8 (47)	4 (44)	18 (46)
**Surgical method, n (%)**				
Laparoscopy or robotic	**11 (85)**	**13 (76)**	**8 (89)**	**32 (82)**
Laparotomy	2 (15)	4 (23)	1 (11)	7 (18)
**Surgery duration, min**	131 ± 37<br>122 [109-143]	146 ± 68<br>137 [101.5-174]	142 ± 55<br>141 [87-175.5]	140 ± 55<br>129 [103-174]

Continuous variables are presented as mean ± standard deviation or median [interquartile range]. ASA, American Society of Anesthesiologists; min, minutes; PACS, physical activity counseling session; SSPP, semi-supervised prehabilitation program; SPP, supervised prehabilitation program.

References

1. Canadian society for exercise physiology (CSEP-PATH). *The CSEP Physical Activity Training for Health: Resource Manual is an evidence-informed, quality textbook and tool for qualified exercise professionals*, 3rd edition ed.
2. Eeckhout, C.F., M. Philippot, P. Auto-efficacité perçue pour la pratique d'une activité physique: adaptation et validation francophone du Exercise Confidence Survey. *Canadian Journal of Behavioural Science/Revue canadienne des sciences du comportement* **2011**.
3. Jones, C.J.; Rikli, R.E.; Beam, W.C. A 30-s chair-stand test as a measure of lower body strength in community-residing older adults. *Res Q Exerc Sport* **1999**, *70*, 113-119, doi:10.1080/02701367.1999.10608028.
4. Webster, K.; Cella, D.; Yost, K. The Functional Assessment of Chronic Illness Therapy (FACIT) Measurement System: properties, applications, and interpretation. *Health Qual Life Outcomes* **2003**, *1*, 79, doi:10.1186/1477-7525-1-79.
5. Roberge, P.; Dore, I.; Menear, M.; Chartrand, E.; Ciampi, A.; Duhoux, A.; Fournier, L. A psychometric evaluation of the French Canadian version of the Hospital Anxiety and Depression Scale in a large primary care population. *J Affect Disord* **2013**, *147*, 171-179, doi:10.1016/j.jad.2012.10.029.
6. Dindo, D.; Demartines, N.; Clavien, P.A. Classification of surgical complications: a new proposal with evaluation in a cohort of 6336 patients and results of a survey. *Ann Surg* **2004**, *240*, 205-213, doi:10.1097/01.sla.0000133083.54934.ae.
7. Borg, G.A. Psychophysical bases of perceived exertion. *Med Sci Sports Exerc* **1982**, *14*, 377-381.
